# COVID-19 Infodemiology: Association Between Google Search and Vaccination in Malaysian Population

**DOI:** 10.7759/cureus.29515

**Published:** 2022-09-23

**Authors:** Ren Yi Kow, Norfazilah Mohamad Rafiai, Akmal Azim Ahmad Alwi, Chooi Leng Low, Muhammad Wafiuddin Ahmad, Zamzuri Zakaria, Ahmad Hafiz Zulkifly

**Affiliations:** 1 Orthopedics, Traumatology, and Rehabilitation, IIUM (International Islamic University Malaysia), Kuantan, MYS; 2 Education and Research, Sultan Ahmad Shah Medical Centre @IIUM (International Islamic University Malaysia), Kuantan, MYS; 3 Plastic and Reconstructive Surgery, Sultan Ahmad Shah Medical Centre @IIUM (International Islamic University Malaysia), Kuantan, MYS; 4 Radiology, Sultan Ahmad Shah Medical Centre @IIUM (International Islamic University Malaysia), Kuantan, MYS

**Keywords:** covid-19, google, infodemiology, malaysia, covid-19 vaccine, google trend

## Abstract

Background

In light of the ongoing coronavirus disease 2019 (COVID-19) pandemic, vaccination is one of the most important defensive strategies in combating the severe acute respiratory syndrome coronavirus 2 (SARS-CoV-2). Vaccine hesitancy or anti-vaccination attitude has become a barrier to the nationwide vaccination program, potentially sabotaging the effectiveness of vaccination. Thus far, Google Trends (GT) has been used extensively for monitoring information-seeking behavior during the pandemic. We aimed to investigate the association between Google search, the vaccination rate, and the number of vaccinated and infected cases among the Malaysian population.

Material and method

GT’s customizable geographic and temporal filters were applied to include results for predetermined keywords from January 1, 2021, to December 31, 2021. Both Malay and English languages were used to reflect the multi-racial and multi-lingual community in Malaysia. The search volume index (SVI) derived was compared with the numbers of vaccinated and infected cases which were extracted from the open-access database (COVIDNOW in Malaysia) within the same period. Both analyses were performed independently by two authors to ensure accuracy of the data extraction process. A descriptive analysis was used to compare GT analyses and the number of daily vaccinations and positive COVID-19 cases.

Results

The information-seeking behavior in the public fluctuated from time to time. The interest surged during the initiation of vaccination program and upon the outbreak of COVID-19 in Malaysia. The surge in interest prior to the peak of vaccination rate also indicated that the public tended to get information online prior to getting the vaccines.

Conclusion

This observational study illustrates the ability of GT to monitor the interest of vaccination among the Malaysian population during the pandemic. By monitoring the dynamic changes in Google Trends, healthcare authorities can get a glimpse of public perceptions such as attitude towards COVID-19 vaccine, hence potentially identify and stymie any dangerous online anti-vaccination rhetoric swiftly.

## Introduction

Since the start of the severe acute respiratory syndrome coronavirus 2 (SARS-CoV-2)-induced coronavirus disease 2019 (COVID-19) pandemic, there has been a monumental toll on the healthcare system [1]. On top of a large number of patients who contracted COVID-19, there is also colossal collateral damage to the healthcare system whereby many patients suffering from non-COVID-19-related diseases have been receiving delayed or sub-standard treatment [1,2]. Thus far, with more than 4.45 million people diagnosed with COVID-19 in Malaysia, there have been 35,583 deaths (28,050 in-patient deaths and 7,533 brought-in-dead) [3].

To defend against COVID-19, the COVID-19 vaccination program was initiated in Malaysia on February 24, 2021 [4,5]. Realizing the importance of vaccine against COVID-19, the Ministry of Health Malaysia has taken steps assiduously to ensure effectiveness of the vaccination program. Based on data published by the Ministry of Health Malaysia, our country has achieved 20% (6.695 million) fully vaccinated rate (at least two doses of vaccines) by July 30, 2021; 40% (13.279 million) by August 22, 2021; and 60% (19.720 million) by September 23, 2021 [4]. In May 2022, 82% (26.795 million) of our population has received at least two doses of COVID-19 vaccines [4].

One of the hurdles faced by the Ministry of Health is vaccine hesitancy or anti-vaccination attitude among a small Malaysian population. Aided by easy access to unverifiable internet sources, the fear of vaccine’s side effects is spreading like a virus. Highlighted by Gunther Eysenbach, one of the four pillars to fight an infodemic is information monitoring (infoveillance) [6]. Ergo, numerous COVID-19-related infodemiological studies have been conducted worldwide to unravel the information-seeking behavior of the public [7-16]. In Malaysia, Google is the main search engine with increasing number of Malaysian population utilizing it to search for information on common medical problems [17]. Similarly, Google Trends analysis reveals that most Malaysians have been seeking information using Google search during this pandemic period [18,19]. Nevertheless, to the best of our knowledge, there is no study investigating the relationship between Google Trends and the vaccination rate.

We aimed to conduct an infodemiological study to find the association between Google search, the rate of vaccination, and the number of infected cases in Malaysia. This study will provide an insight into whether the number of infected cases and the rate of vaccination in Malaysia are influenced by the search behavior of the Malaysian population.

## Materials and methods

There were two parts in this study. The first part included an analysis of Google Trends (GT) while the second part involved an analysis of vaccination rate among the Malaysian population via an open-access database. Both analyses were performed independently by two authors to ensure accuracy of the data extraction process. Any discrepancy was resolved by re-evaluation of GT or open-access database analyses by the study team.

In the first part of GT analysis, GT’s customizable geographic and temporal filters were applied as outlined by Mavragani [20]. The search results were specific to Malaysia from January 1, 2021, to December 31, 2021. Keywords such as “vaccine covid19” and “vaccine booster” were used for GT analysis. Bearing the geographical influence of languages used in Google search, we also used Malay language keywords such as “vaksin covid19” and “vaksin penggalak” for the GT analysis [17]. The search volume index (SVI) derived from using the above keywords within the study duration was documented. The search volume index (SVI) obtained via the GT was scaled from 0 to 100 within the search duration [9]. 

In the second part, the number of individuals receiving vaccines in Malaysia was obtained from the open-access database (COVIDNOW in Malaysia) [4]. The number of documented vaccinations per day was extracted and charted into a graph together with SVI of GT analysis. The number of daily positive COVID-19 cases was also extracted from the database [4]. A descriptive analysis was used to compare GT analyses with the number of daily vaccinations and positive COVID-19 cases.

## Results

COVID-19 vaccines

**Figure 1:**
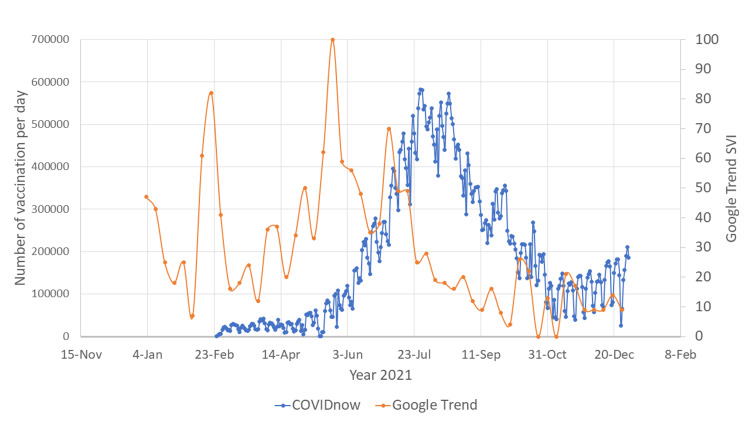
Google Trend word search "vaccine covid19" vs COVIDnow data SVI: search volume index; COVID-19: coronavirus disease 2019

**Figure 2:**
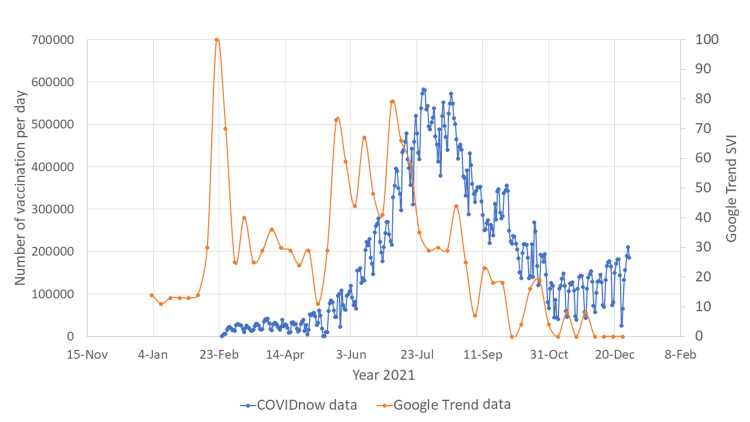
Google Trend search word "vaksin covid19" vs COVIDnow data SVI: search volume index; COVID-19: coronavirus disease 2019

There was a surge in SVI for both English and Malay language searches for COVID-19 vaccines in mid-February 2021 (Figures 1, 2). This surge coincided with the date of initiation of the COVID-19 vaccination program in Malaysia [4].

**Figure 3:**
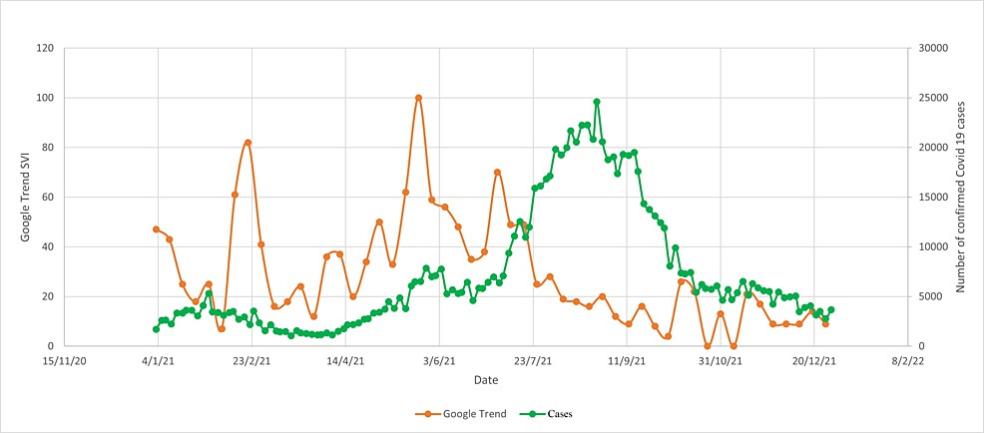
Google trend word search "vaccine covid19" vs confirmed COVID-19 cases SVI: search volume index; COVID-19: coronavirus disease 2019

**Figure 4:**
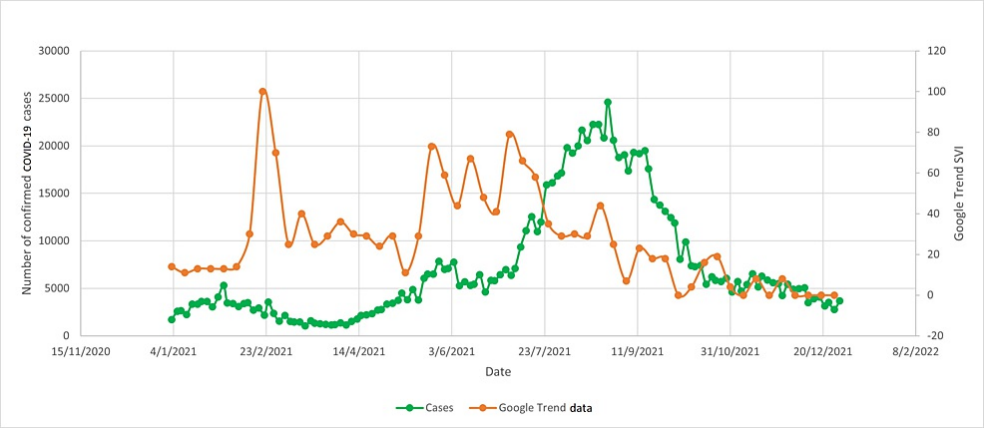
Google trend search word "vaksin covid19" vs confirmed COVID-19 cases SVI: search volume index; COVID-19: coronavirus disease 2019

There was another surge in Google search for COVID-19 vaccination between June 2021 and July 2021, possibly related to the increasing number of people infected with COVID-19 during that period (Figures 3, 4). In this duration, the vaccination rate in Malaysia also increased exponentially and reached a peak in August 2021. Surges in Google search for vaccines during February 2021 and June 2021 were observed in both English and Malay languages.

Vaccine booster

**Figure 5:**
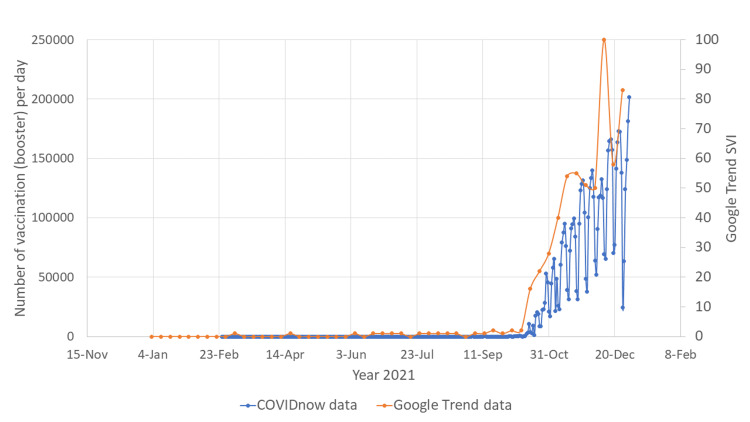
Google Trend search word "vaksin booster" vs COVIDnow data SVI: search volume index; COVID-19: coronavirus disease 2019

**Figure 6:**
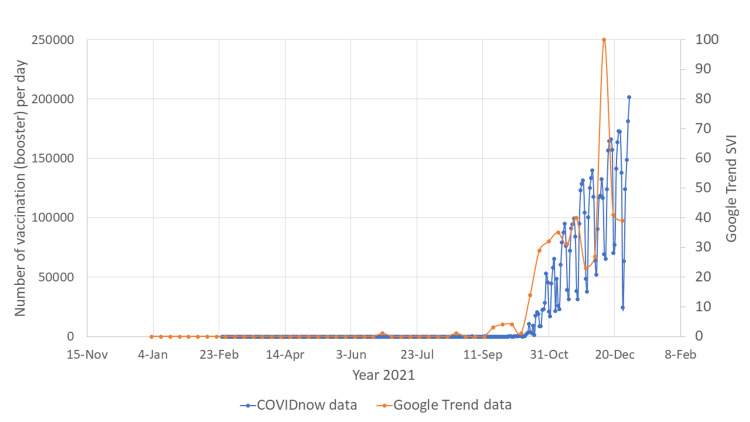
Google Trend search word "vaksin penggalak" vs COVIDnow data SVI: search volume index; COVID-19: coronavirus disease 2019

COVID-19 booster vaccination was implemented in Malaysia since September 2021. Since the announcement of COVID-19 booster vaccine being inaugurated, there was a steadfast increase in Google search for COVID-19 vaccine booster (Figures 5, 6). This trend was almost matching for both English and Malay language Google searches. The increasing Google searches for COVID-19 vaccine booster reached a peak just before the end of the year 2021, and it coincided with the highest number of people receiving COVID-19 booster doses.

## Discussion

Considering the ongoing COVID-19 pandemic, this is the first study in Malaysia that investigates the behavior of public interest in COVID-19 vaccination using GT analysis. A 10-year GT analysis by Kow et al. reveals that GT is a viable platform to track healthcare interest among the Malaysian population, despite a geographical variation in the language used [17]. Similar behavior has been noted by Izhar and Torabi and Lim et al. whereby the Malaysian public has been using Google search extensively to enquire information related to COVID-19 during the pandemic [18,19]. 

There were two major surges of the number of Google searches for COVID-19 vaccines (Figures 1, 2). The first surge happened in mid-February 2021, when the Malaysian government first launched the COVID-19 vaccination program. This indicates that the Malaysian public was actively looking for information on the vaccines to help them make the decision whether to receive the vaccines or not. The first surge of the number of Google searches for vaccine was also preceded by an outbreak of COVID-19 infection (Figures 3, 4). Due to the sheer number of confirmed COVID-19 infected cases and the launching of COVID-19 vaccination program, Malaysian public resorts to the most easily accessible information online to know more about the COVID-19 vaccine. The second surge occurred in June and July 2021, just before mass vaccination of the Malaysian population took place. Similar to the first surge, the public also turns to Google search engine for reassurance prior to the vaccination. Mimicking the public behavior of using Google search prior to vaccination, the number of Google search for the vaccine booster also raised in conjunction with the increasing number of people who were having vaccine booster (Figures 5, 6). 

Characterization of information-seeking behavior via Google searches is crucial and it may be helpful in planning by healthcare authorities. GT has been found to be able to predict COVID-19 outbreaks in India and Italy [10,11]. In this study, we have demonstrated that there is an association between Google search, vaccination rate, and number of infected cases. The association exists on both COVID-19 vaccine and vaccine booster. The trend is almost identical for those terms in Malay and English languages, indicating that Malaysians generally use Google search to seek information, irrespective of their level of education. By utilizing GT as a surrogate tool to monitor the dynamic change in information-seeking behavior among Malaysians, the authorities can predict and mobilize crucial resources to on-demand areas. By monitoring the GT, government administrators can always keep their finger on the pulse of public curiosity. By identifying a surge in a key area, the administrators can launch a campaign to explain and alleviate any doubt the public may have. 

Limitations

This study has several limitations. First and foremost, this study uses Google Trends which only analyses the search behavior of people using the Google search engine. Nevertheless, as previous studies have indicated, Google is an overwhelming search engine of choice among Malaysians, hence the risk of bias will be kept to a minimum [17]. In addition, keywords used in GT analysis will affect SVI. For example, keyword such as “vaccine” revealed a bigger SVI compared to “vaccine covid 19,” as the former keyword is non-specific and may reveal searches for vaccines of other diseases, albeit in a smaller proportion during the pandemic. In contrast, keywords such as “vaccine booster” and “vaksin penggalak” are chosen for GT analyses instead of keywords such as “vaccine booster covid 19” and “vaksin penggalak covid 19” owing to inadequate data on GT for these keywords. Besides that, a descriptive interpretation is adopted in this study as dynamic changes within the timeline make statistical analysis impractical. As such, all the findings presented in this study may be correlational, but the causality cannot be assessed. Lastly, we do not include complex statistical analysis as illustrated by Rovetta for better reading and understanding by the general public [21].

## Conclusions

This study augments the belief that the Malaysian population routinely uses Google search to obtain information on COVID-19 vaccines. Nevertheless, there is a dynamic change in the interest based on timing and the number of infected cases. By monitoring Google Trends, healthcare planners in Malaysia can get a glimpse of public perceptions such as attitudes toward COVID-19 vaccines. In the same vein, by tracking Google Trends, healthcare authorities can quickly identify and stymie any dangerous online anti-vaccination rhetoric.
